# Contributions of Roy J. Shephard to the Study of Circumpolar Human Biology and Health

**DOI:** 10.1002/ajhb.70123

**Published:** 2025-08-20

**Authors:** William R. Leonard, Peter T. Katzmarzyk

**Affiliations:** ^1^ Department of Anthropology, Program in Global Health Studies Northwestern University Evanston Illinois USA; ^2^ Robert J. Havey, MD Institute for Global Health, Feinberg School of Medicine, Northwestern University Chicago Illinois USA; ^3^ Pennington Biomedical Research Center Baton Rouge Louisiana USA

**Keywords:** circumpolar health, energetics, human adaptability, Inuit, metabolism

## Abstract

More than any other scholar in our field, Professor Roy J. Shephard's research has shaped and transformed our understanding of the biology and health of circumpolar populations. His long‐term research among the Inuit of Igloolik, Canada has provided the field of human biology with foundational insights into how human populations adapt to arctic climates, and how the transition to a market‐oriented lifestyle erodes fitness and metabolic health. Shephard was the prime architect of early research done in the Canadian Arctic as part of the Human Adaptability Program (HAP) of the International Biological Programme (IBP) in the 1960s and early 1970s. After the original IBP studies, Shephard and collaborator Andris Rode continued their research in Igloolik through the early 1990s. This long‐term research provided some of the first clear evidence on how the process of acculturation and lifestyle change erodes physical development and metabolic health among Indigenous populations of the north. This paper provides an overview of the major findings and insights from Roy Shephard and colleagues' research in Igloolik and highlights how these contributions are shaping ongoing research on the biology and health of circumpolar populations.

## 1 Introduction

The biology of Indigenous arctic (circumpolar) populations has long been a focus of research in human population biology. Circumpolar populations have adapted to severe climatic and ecological stressors that dramatically shape their metabolism, growth, and dietary patterns, influencing both physical and mental well‐being. Today, ongoing social and environmental changes are eroding the health of circumpolar peoples and threatening their livelihoods.

Current work in human biology attempts to better understand the health and adaptive challenges faced by arctic populations in order to fashion sustainable and resilient strategies for the future. These new research directions build upon the foundational work launched over 60 years ago as part of the Human Adaptability Program (HAP) of the International Biological Programme (IBP). The IBP‐HAP was initiated in 1964 and provided support for some of the first large‐scale, comparative research studies on human biological variation and adaptability to ecological stressors (see Leonard 2018; Little and Collins 2012; Weiner 1964, 1977).

**FIGURE 1:**
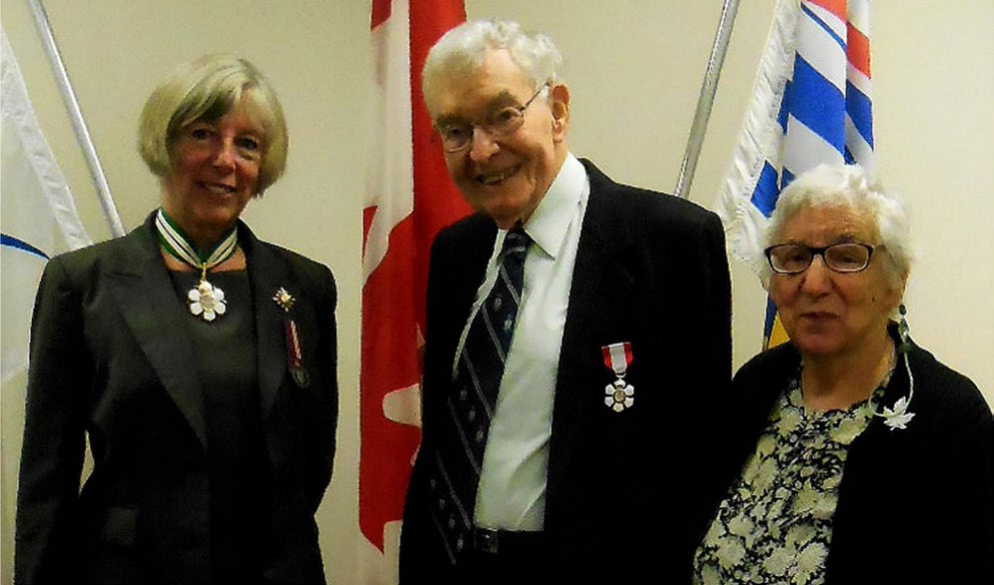
Roy J. Shephard (center), with his wife Muriel (right), being appointed to the Order of Canada, May 18, 2014. *Source:* Photo courtesy of Sarah Shephard.

The prime architect of the research done in the Canadian Arctic as part of the IBP‐HAP was Professor Roy J. Shephard (1929–2023) of the University of Toronto. Professor Shephard was a prolific scholar whose work shaped many areas of research, including human biology, exercise science, physical activity, and public health (Katzmarzyk and Bouchard 2023). Over the span of his career, Dr. Shephard published over 2000 refereed papers and over 100 books. As a faculty member at the University of Toronto from 1964 to 1994, Shephard trained over 30 masters and doctoral students and mentored 11 postdoctoral fellows. In May of 2014 he was appointed to the Order of Canada “for his pioneering work in the field of exercise science and for promoting the health benefits of physical activity to Canadians” (see Figure 1).

**FIGURE 2:**
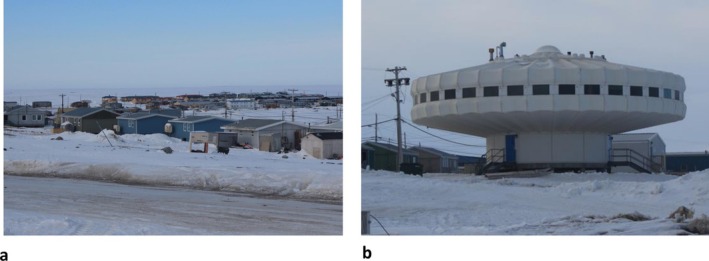
(a) Community of Igloolik in the Nunavut Territory of Canada. (b) The Igloolik Research Center, site for much of Professor Roy Shephard's research on the biology and health of the Inuit. *Source:* Photos courtesy of Tracey Galloway.

Within human biology, Roy Shephard is best known for his long‐term research among the Inuit of the arctic community of Igloolik in the Nunavut Territories of Canada (see Figure 2). More than any other scholar in our field, Shephard's research has shaped and transformed our understanding of the biology and health of circumpolar populations.

Shephard's initial IBP‐sponsored work in Igloolik ran from 1967 to 1974. This research provided the first comprehensive assessment of physiological adaptability and health among an indigenous circumpolar group. Data collected as part of the IBP research included: (a) anthropometric assessment of growth, body size, and body composition (Rode and Shephard 1973a; Shephard 1974; Shephard et al. 1973), (b) measurement of energy expenditure and physical activity (Godin and Shephard 1973), (c) assessment of aerobic capacity and physical strength (Rode and Shephard 1971; Shephard 1974), and (d) evaluation of cardiovascular health (Rode and Shephard 1971, 1973b, 1973c).

After the original IBP studies, Shephard and collaborator Andris Rode returned to Igloolik in 1979–1981 (Rode and Shephard 1984a, 1984b) and again in 1989–1990 (Rode and Shephard 1994a, 1994b) to study and document the changes in physiology and health associated with the transition from a subsistence lifeway to a more sedentary, market‐oriented lifestyle. Their longitudinal work, summarized in the 1996 book, *The Health Consequences of ‘Modernization’: Evidence from Circumpolar Peoples*, provided some of the first clear evidence into how the process of acculturation and lifestyle change erodes physical development and metabolic health among Indigenous populations of the north (Shephard and Rode 1996).

Roy Shephard's generous input and guidance were very helpful when we were setting up our research on the biology and health of Indigenous Siberian populations in the early 1990s (Crawford et al. 1992; Katzmarzyk 1993; Katzmarzyk et al. 1994a, 1994b; Leonard et al. 1992, 1994). Indeed, much of the research on the biology and health of Indigenous circumpolar populations over the last 30 years has built upon the pioneering work that Shephard and colleagues carried out in Igloolik.

In this paper, we first provide an overview of the major findings and insights from Roy Shephard and colleagues' long‐term research in Igloolik. We then consider how our own work among Indigenous Siberian populations has built upon Shephard's contributions to our understanding of the biology and health of circumpolar populations.

## 2 Contributions of Roy Shephard to Circumpolar Human Biology and Health

### 2.1 Early Research in Igloolik: The IBP‐HAP Years, 1967–1974

The early research by Roy Shephard and colleagues as part of the IBP‐HAP provided some of the first population data on the biology, health, and adaptive strategies of Indigenous populations of the Arctic. A central component of all the IBP‐HAP research was the establishment of standardized methodologies for assessing key aspects of human population biology, such as (a) growth and development, (b) health and nutritional status, (c) reproduction and demography, (d) energy expenditure and working capacity, and (e) genetic variation (see Shephard 1978; Shephard et al. 1968; Weiner and Lourie 1969, 1981).

Two of Roy Shephard's graduate students, Andris Rode and Gaétan Godin, played important roles in the early Igloolik research. Rode (1972) conducted his dissertation research in Igloolik, studying the cardiorespiratory fitness and strength of the Inuit. Godin (1972) studied patterns of energy expenditure and activity levels among Igloolik Inuit for his MSc thesis. Rode continued to collaborate on the subsequent research in Igloolik through the 1990s.

Some of the major research findings of the IBP‐HAP Studies in Igloolik included the following: (a) more traditionally‐living Inuit had elevated basal metabolic rates (BMR) and very high levels of energy expenditure and physical activity (Godin and Shephard 1973; Rode and Shephard 1995), (b) high aerobic capacity (VO_2_max) and physical strength, on par with trained athletes (Rode and Shephard 1971), (c) low body fat and lean physiques, despite having relatively high weight‐for‐height ratios (Shephard et al. 1973), and (d) excellent cardiovascular health despite consuming diets high in meat and fat (Rode and Shephard 1973b, 1973c).

**FIGURE 3:**
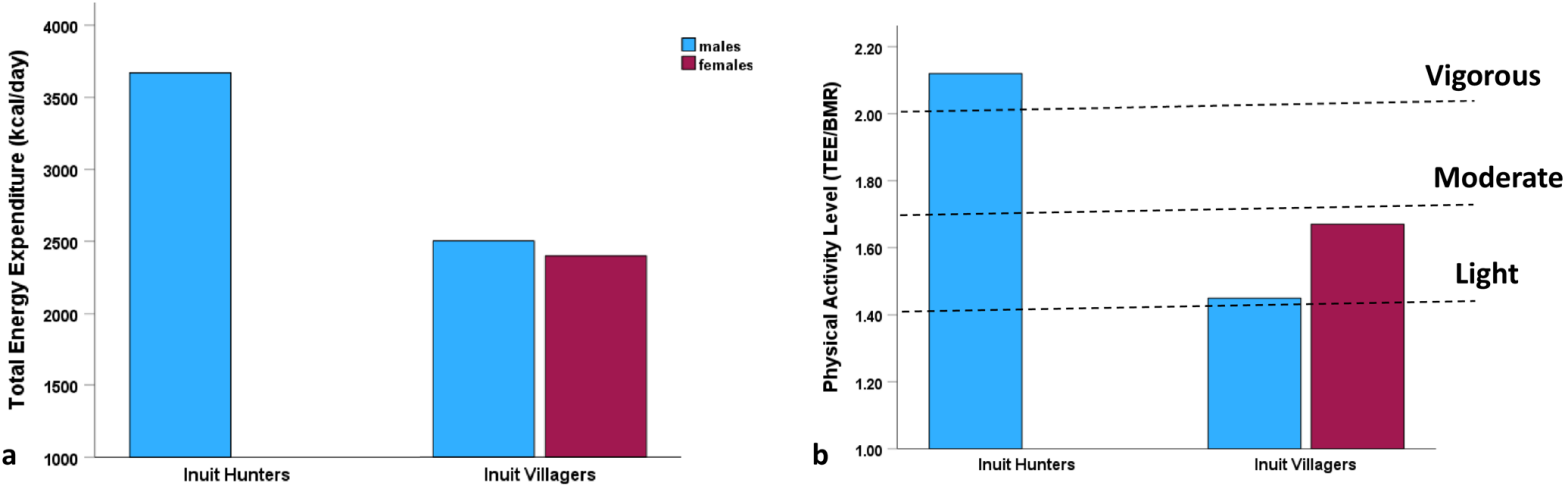
(a) Total energy expenditure (TEE; kcal/day) and (b) physical activity levels (PAL; TEE/BMR) among Igloolik Inuit hunters and villagers, 1969–1971. Inuit hunters have very high levels of energy expenditure and physical activity (3670 kcal/day and a PAL of 2.12). Inuit villagers have much more modest activity levels (PALs: 1.45 for men and 1.67 for women). *Source:* Data from Godin and Shephard (1973) and WHO (2004).

Figure 3 shows the results of Godin and Shephard's (1973) study of total energy expenditure (TEE; kcal/day) and physical activity levels (PAL; TEE/BMR). Inuit hunters had expenditure levels of over 3600 kcal/day, with a PAL in the “vigorous” range based on current WHO (2004) guidelines. In contrast, Inuit villagers had more modest expenditure and activity levels in the “light” to “moderate” ranges, with PALs of 1.45 and 1.67 for men and women, respectively.

**FIGURE 4:**
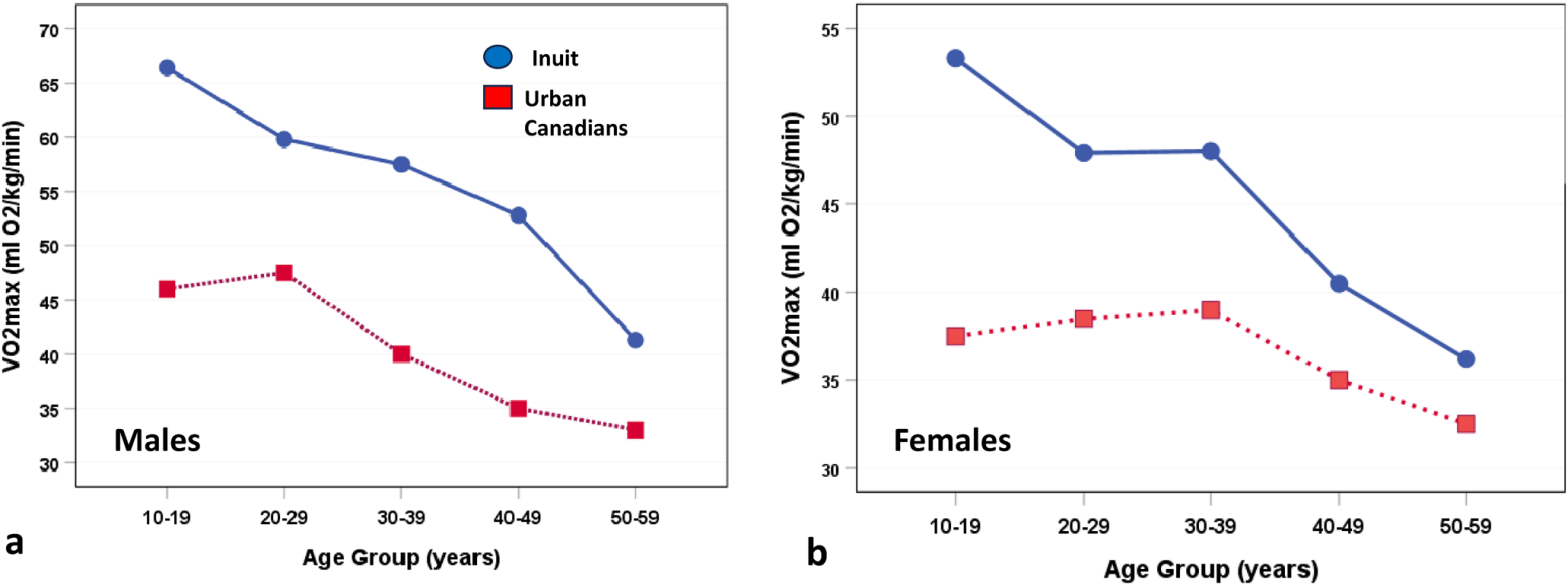
Comparison of maximal aerobic capacity (VO_2_max; mL O_2_/kg/min) in Igloolik Inuit and urban Canadian (a) men and (b) women, 1969–1970. Across all age groups, Inuit men and women have substantially higher aerobic capacities than their urban Canadian peers from Toronto. *Source:* Data from Rode and Shephard (1971).

Figure 4 shows results from Rode and Shephard's (1971) study comparing VO_2_max (mL O_2_/kg/min) of the Igloolik Inuit to their urban Canadian peers measured in Toronto. Across the entire age span, the Inuit males and females had significantly higher aerobic capacities than their urban counterparts (*p* < 0.01). Additionally, this study found that Inuit leg strength was 50% greater than that of their urban peers.

**FIGURE 5:**
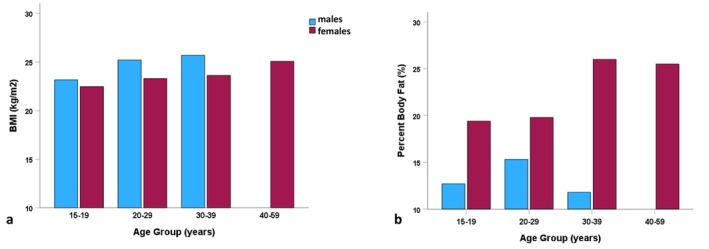
(a) Body mass indexes (BMI; kg/m^2^) and (b) percent (%) body fatness among Igloolik Inuit ages 15–59 years, 1970. For the entire sample, mean BMIs were 24.7 kg/m^2^ in men and 23.7 kg/m^2^ in women. Percent body fatness averaged 13.4% in men and 22.6% in women. *Source:* Data from Shephard et al. (1973).

Figure 5 presents the results of Shephard et al.'s (1973) study of body mass and body composition among the Inuit. Body composition in the study was measured using deuterium isotope dilution. Note that while the body mass indexes (BMI; kg/m^2^) of the Inuit fall near or above the threshold for “overweight” (BMI = 25 kg/m^2^), the adiposity levels are quite low, with percent body fat averaging 13% in men and less than 23% in women. These findings are notable in being among the first to highlight the limitations of weight‐for‐height indices in assessing overweight and obesity, a topic that continues to be discussed and debated in public health and medicine today (see Rubino et al. 2025).

The IBP‐era studies in arctic communities of North America and Eurasia all concluded by the mid‐1970s. The findings from these different studies were summarized in a landmark volume edited by F.A. Milan (1980), *The Human Biology of Circumpolar Populations*. Roy Shephard's chapter in this volume summarized the research from Igloolik. He concluded this chapter with a clear indication of his future research directions, noting:

It will be of great interest to repeat these physiological studies in a few years time, when circumpolar communities have made further ‘progress’ towards the urban life‐style. (Shephard 1980, 337)

### 2.2 Igloolik Research: 1979–1981

By the 1980s, research on adaptability and health among arctic populations was largely abandoned in human biology, leaving us with what Ted Steegmann (2007) characterized as an “unfinished agenda” in his Raymond Pearl Memorial Lecture to the Human Biology Association in 2006. Roy Shephard and Andris Rode were among the very few human biologists to continue with their IBP research in the arctic, conducting follow‐up studies in Igloolik in 1979–1981 and 1989–1990. This work explored the profound health and nutritional consequences of acculturation, lifestyle change, and environmental pollution/degradation in the Canadian arctic.

**FIGURE 6:**
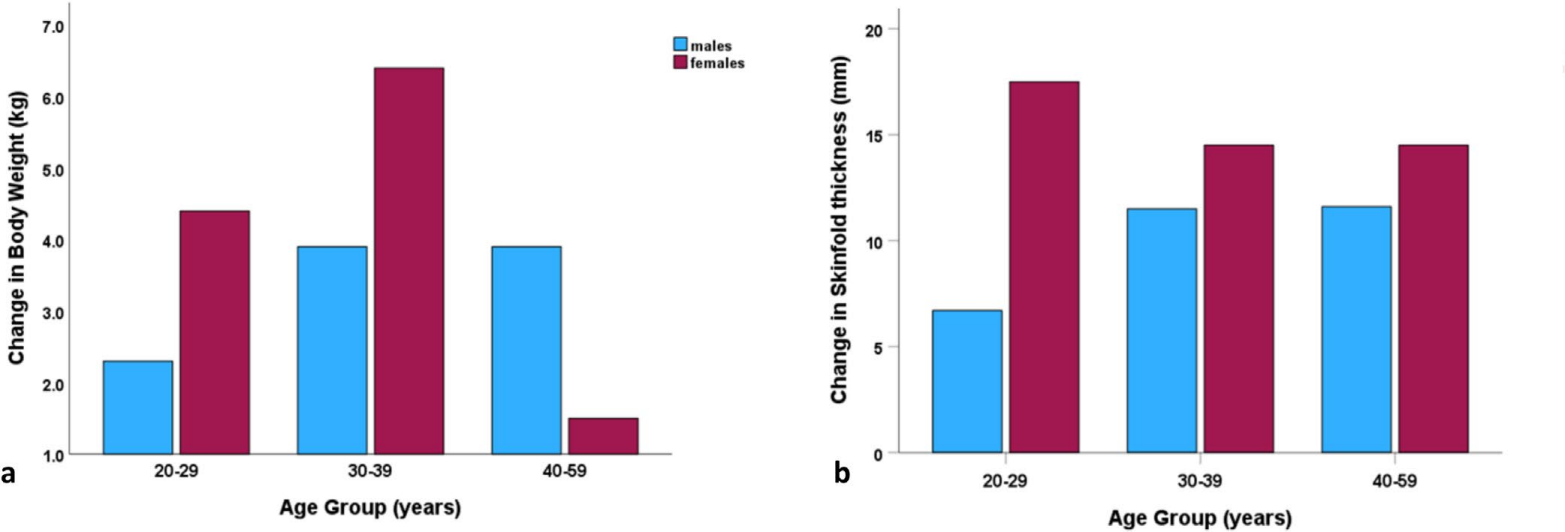
Changes in (a) body weight (kg) and (b) sum of three skinfolds (mm) among Igloolik Inuit males and females between 1970–1971 and 1980–1981. Men gained an average of 3.4 kg, whereas women gained an average of 4.1 kg (*p* < 0.01). Gains in adiposity were more dramatic, with men gaining an average of 9.9 mm and women gaining an average of 15.5 mm (*p* < 0.01). *Source:* Data from Rode and Shephard (1984a).

By the early 1980s, their research in Igloolik was documenting: (a) marked declines in aerobic fitness, (b) increasing body weights and adiposity, and (c) dramatic reductions in physical strength (Rode and Shephard 1984a, 1984b). Figure 6 shows the changes in body weight (kg) and the sum of three skinfolds (mm) among Igloolik adults between 1970–1971 and 1980–1981 (from Rode and Shephard 1984a). On average, Igloolik men and women gained 3–4 kg in body weight (*p* < 0.05), while skinfold measures increased by almost 10 mm in men and 15.5 mm in women (*p* < 0.01). This study also documented a 15% reduction (*p* < 0.05) in aerobic capacity, along with significant declines in leg strength (*p* < 0.05).

### 2.3 Igloolik Research: 1989–1990

In their third wave of research in 1989–1990, Shephard and Rode found further erosion of the health and fitness of the Igloolik Inuit. There was little evidence of a secular trend in stature during this time. In contrast, there were significant increases in both body weight and fatness over the two decades. Additionally, this work also documented significant declines in aerobic capacity and grip strength (Rode and Shephard 1994a).

**FIGURE 7:**
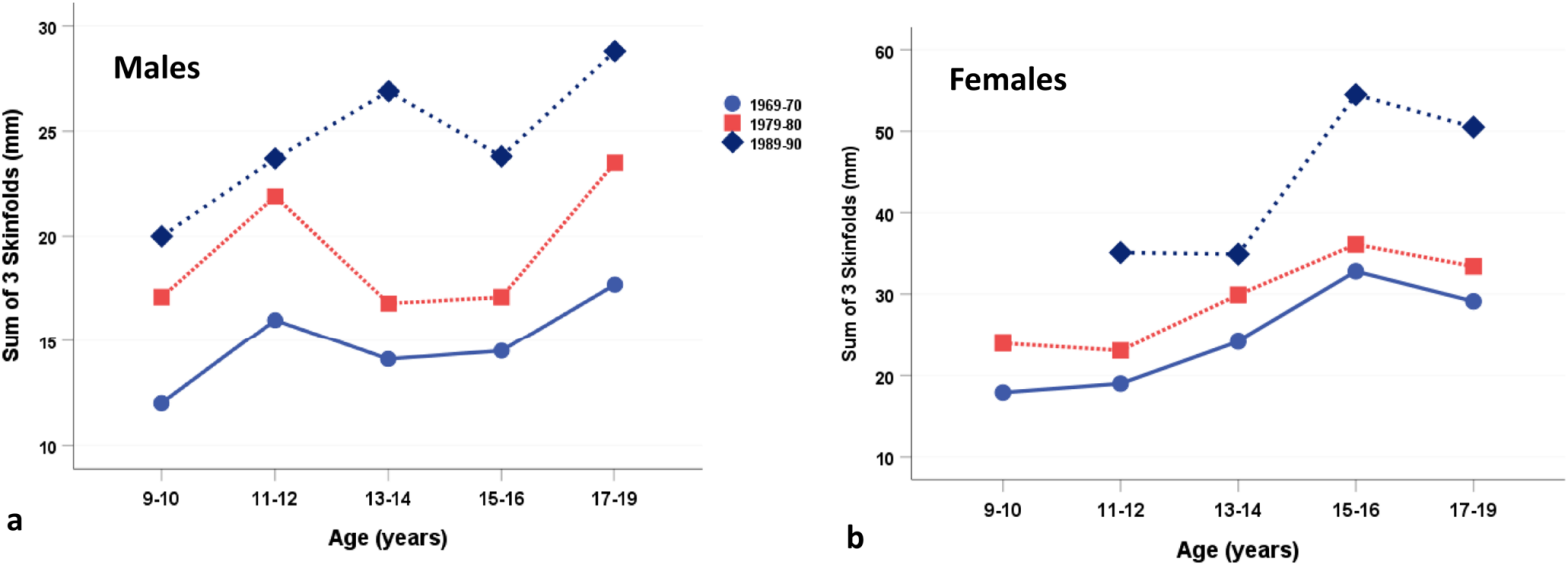
Sum of three skinfolds (mm) in 1969–1970, 1979–1980, and 1989–1990 among Igloolik Inuit (a) males and (b) females, ages 9–19 years. Both males and females show substantial and significant increases in adiposity over the three survey waves (*p* < 0.01). *Source:* Data from Rode and Shephard (1994a).

Figure 7 shows the marked increases in the sum of three skinfolds (mm) among Igloolik children and adolescents between the 1969–1970 and 1989–1990 surveys (Rode and Shephard 1994a). Both boys and girls showed systematic and significant increases in adiposity across the three survey waves (*p* < 0.01).

**FIGURE 8:**
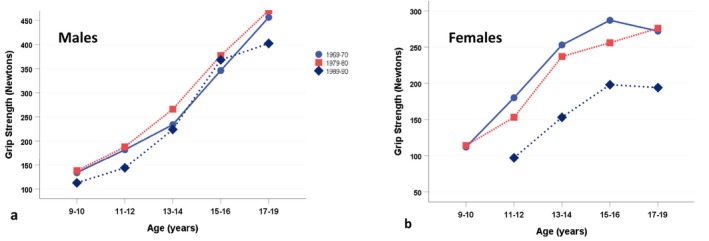
Grip strength (N) in 1969–1970, 1979–1980, and 1989–1990 among Igloolik Inuit (a) males and (b) females ages 9–19 years. Among males, grip strength was generally maintained between 1969–1970 and 1979–1980 but declined between 1979–1980 and 1989–1990 (*p* < 0.05). Igloolik females showed modest declines in grip strength between 1969–70 and 1979–80, and more dramatic declines between 1979–80 and 1989–90 (*p* < 0.01). *Source:* Data from Rode and Shephard (1994a).

Figure 8 shows the changes in grip strength (Newtons) among Igloolik children and adolescents across the three survey periods (Rode and Shephard 1994a). Among boys, grip strength was generally maintained between 1969–1970 and 1979–1980 but declined between 1979–1980 and 1989–1990 (*p* < 0.05). Igloolik girls showed modest declines in grip strength between 1969–1970 and 1979–1980, and more dramatic declines between 1979–1980 and 1989–1990 (*p* < 0.01).

**FIGURE 9:**
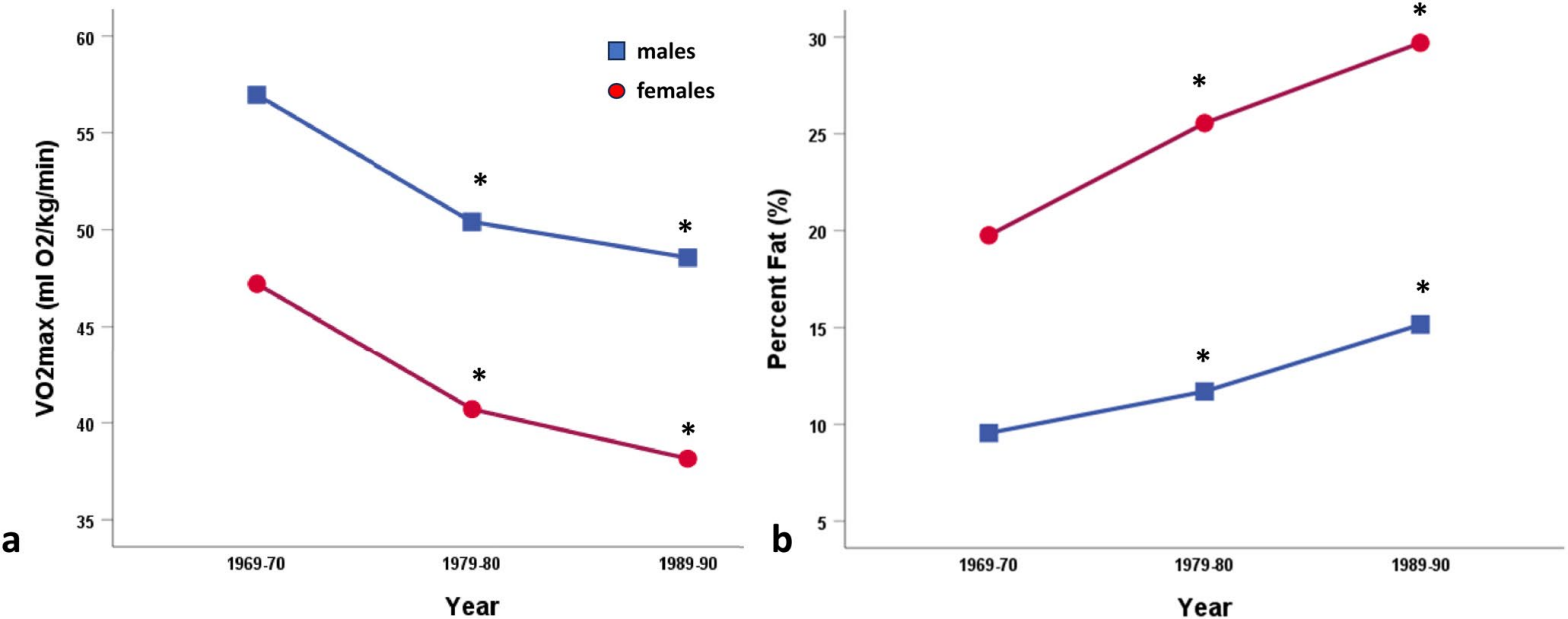
(a) VO_2_max (mL O_2_/kg/min) and (b) percent (%) body fat measures for Igloolik Inuit adults (ages 20–39 years) measured in the years: 1969–1970, 1979–1980, and 1989–1990. In both males and females there are significant declines in maximal aerobic capacity and significant increases in adiposity over time (*p* < 0.05). *Source:* Data from Rode and Shephard (1994b). Percent body fatness estimated from sum of skinfolds using the Durnin and Womersley (1974) equations.

Similar trends were observed among Igloolik adults. Both men and women showed further declines in aerobic fitness and strength, while showing significant increases in body weight and adiposity. Figure 9 highlights the dramatic and significant declines in aerobic capacity between 1970 and 1990 (*p* < 0.01), contrasted with the significant increases in percent body fatness over the same time period (*p* < 0.01) (Rode and Shephard 1994b).

With the end of their Igloolik research in the early 1990s, Rode and Shepard offered the following conclusions from their work and recommendations for promoting health and fitness in the community. They noted that:

As in many other indigenous populations, a loss of the traditional physically active lifestyle has led to a rapid deterioration in health‐related fitness among the circumpolar Inuit of Igloolik. (Rode and Shephard 1994b, 523)

Further, they believe that a return to “traditional patterns of hunting, trapping and fishing no longer seems a viable option” for promoting fitness and health in Igloolik (Rode and Shephard 1994b, 523). Rather, they propose the development of active programs of leisure among the Igloolik Inuit to help restore fitness and improve cardiovascular health.

## 3 Continued Research on Circumpolar Human Biology and Health in Siberia: 1991–Present

Just as Shephard and Rode were completing their research in Igloolik in the early 1990s, we were initiating our work on the biology and health of Indigenous populations of Siberia. Our work in Siberia has been an international collaboration that has focused on two main research questions. First, there is the question of how Indigenous Siberian populations adapted to the cold and marginal climate: do we find evidence of metabolic adaptations in response to these extreme environments? Second, we are interested in how ongoing social‐economic changes in Post‐Soviet Russia are influencing the health and nutrition of Indigenous Siberians.

To address these questions, we have worked with several different Siberian populations over the last 30 years: (a) the Evenki reindeer herders and Ket fishers of Central Siberia, (b) the Buryat cattle herders of the southern Siberian steppes, and (c) since 2003, longitudinal research among the Yakut cattle and horse herders of the northeastern Siberian boreal forest.

**FIGURE 10:**
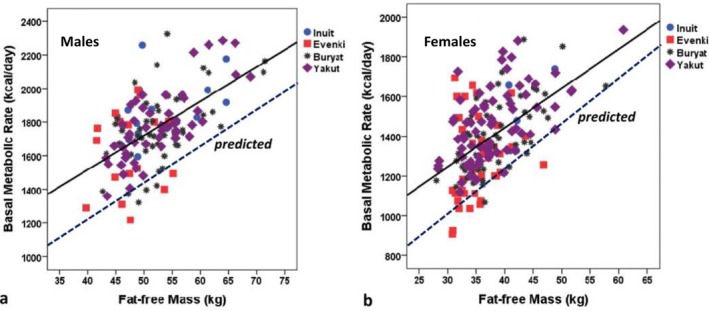
Basal metabolic rate (kcal/day) versus fat‐free mass (FFM; kg) among adult (a) males and (b) females from Indigenous arctic populations (North American: Inuit; Siberian: Evenki, Buryat, Yakut). The solid lines denote the best fit regression lines for BMR versus FFM, while the dotted lines denote the predicted BMR values based on the equation of Cunningham (1991). Measured BMR values are significantly greater than those predicted based on FFM in both men (+18%) and women (+20%), indicating greater rates of metabolic heat production in these cold‐adapted populations. *Source:* Adapted from Leonard (2018).

This research has shown that both the Inuit and Indigenous Siberians show significant and systematic elevations in BMR compared to international reference standards. Figure 10 shows the relationship between BMR and fat‐free mass (FFM; kg) among Igloolik Inuit and Siberian men and women. The solid regression lines for the Indigenous northern groups are significantly elevated above the dashed reference lines in both men and women. Indigenous men, on average, have BMRs that are 18% above reference values, whereas women deviate by +20% (*p* < 0.001).

**FIGURE 11:**
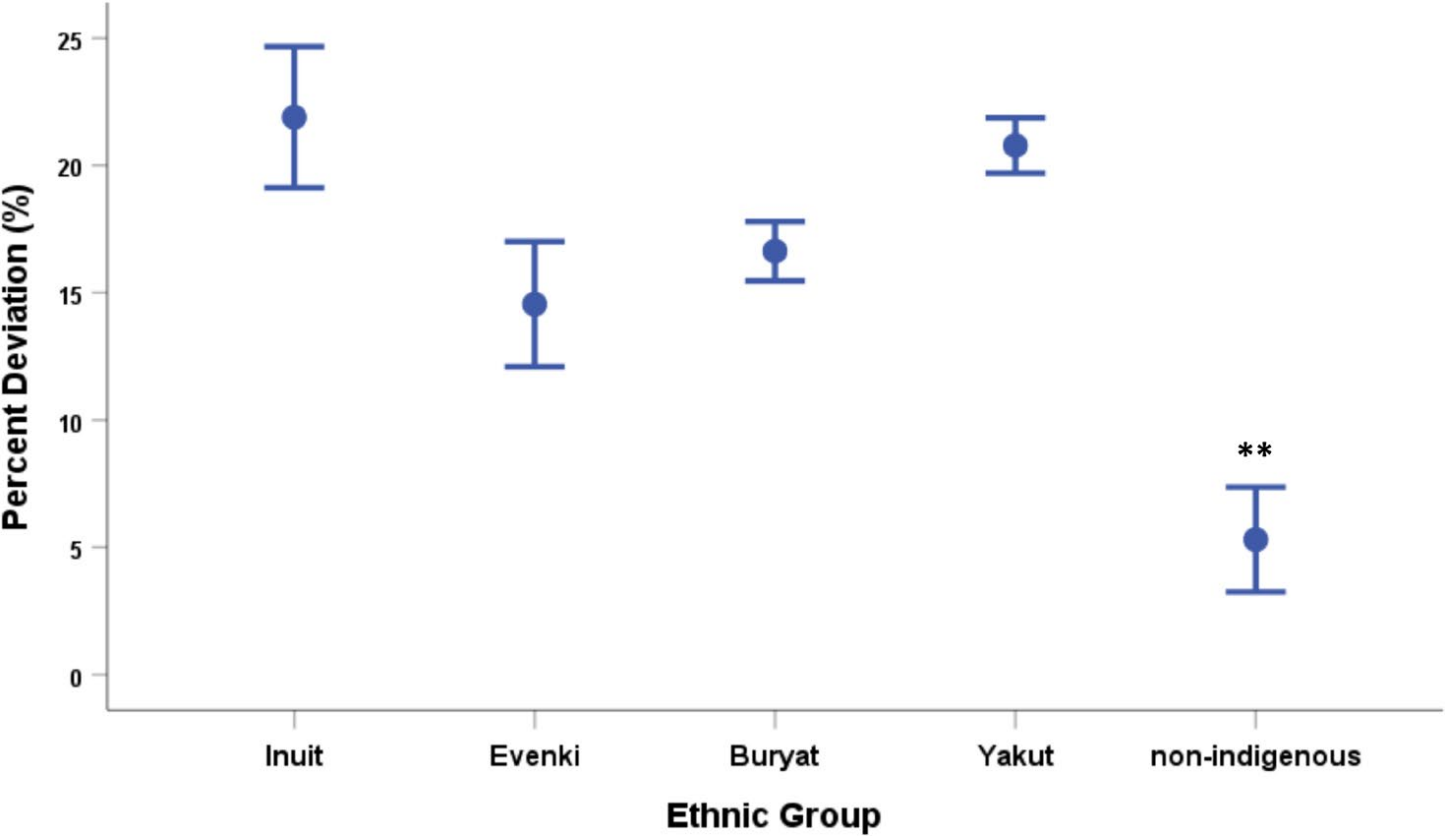
Mean (±SE) deviations from predicted basal metabolic rates (BMR) among indigenous and nonindigenous circumpolar groups. Elevations in BMR among subjects from each of the Indigenous populations (Inuit: +22%; Evenki: +15%; Buryat: +17%; Yakut: + 21%) are significantly greater than that of their nonindigenous counterparts (+5%). **p < 0.01.

Figure 11 shows the percent deviation from predicted BMRs for each of the four Indigenous Arctic populations and nonindigenous individuals living in the same Arctic communities. The Indigenous groups each have BMRs that are elevated by between 15% and 22%. Moreover, the deviations in each of the Indigenous groups are significantly greater than the 5% elevation observed in the nonindigenous subjects (*p* < 0.01). These results suggest that increased metabolic rates may reflect genetic and/or developmental adaptations.

There appear to be at least three major pathways for promoting increased metabolic heat production among Indigenous Siberians. These include: (a) alterations in thyroid function, with enhanced uptake of thyroid hormones in the winter cold (Leonard et al. 1999, 2014; Levy et al. 2013), (b) significant levels of active brown adipose tissue among the Yakut, promoting enhanced non‐shivering thermogenesis in response to cold (Levy et al. 2022, 2021, 2018), and (c) a distinct set of mutations in the mitochondrial genome that are linked to greater uncoupling of oxidative phosphorylation and increased metabolic heat production (Mishmar et al. 2003; Ruiz‐Pesini et al. 2004; Zlojutro et al. 2008).

**FIGURE 12:**
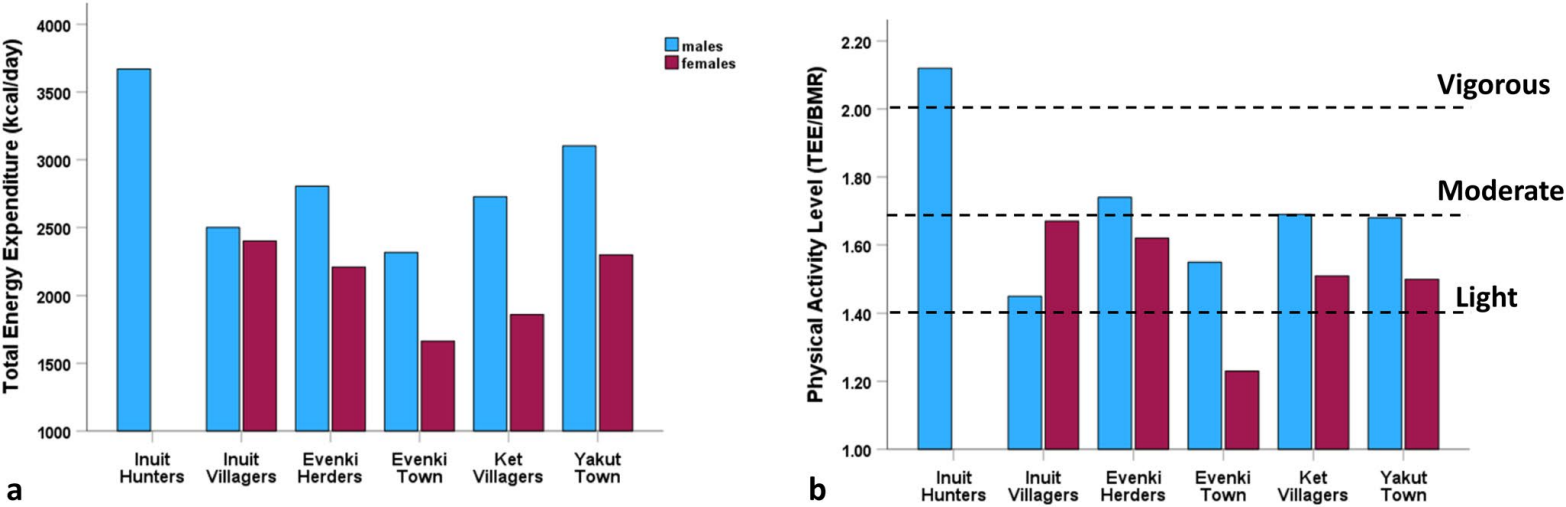
(a) Total energy expenditure (TEEE; kcal/day) and (b) physical activity levels (PAL; TEE/BMR) among Igloolik Inuit (1970–71), Siberian Evenki (1992, 1995), Ket (1992), and Yakut (2003). Among the Siberian groups, Evenki living in the small herding encampments had the highest PALs, in the moderate activity range, still well below the activity levels of the Igloolik hunters. The lowest activity levels were observed among town‐dwelling Evenki. *Source:* Data from Leonard et al. (2005) and WHO (2004).

We have also measured levels of energy expenditure and physical activity among Indigenous Siberians living in more traditional and more urbanized settings. Figure 12 compares TEE and PALs of the Igloolik Inuit to those of the Evenki, Ket, and Yakut. Of the Siberian groups, the Evenki living in the small herding encampments had the highest PALs (1.74 for men and 1.62 for women), in the moderate activity range, still well below the activity levels of the Igloolik hunters. The lowest activity levels were observed among Evenki living in the town of Baykit (PALs of 1.55 for men and 1.23 for women). Among the Siberian groups, sex differences in energy expenditure and activity levels are more marked in the village and town dwellers than in the herding encampments. Additionally, in the Siberian populations, activity levels are consistently higher in men, whereas the opposite is true for the Igloolik villagers.

**FIGURE 13:**
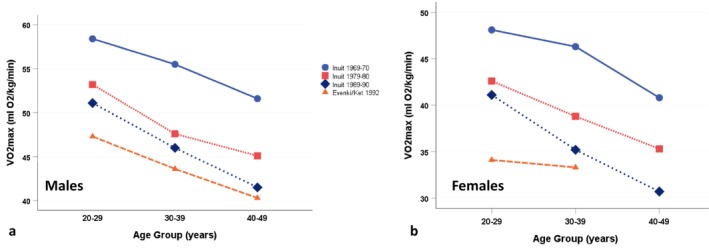
VO_2_max (mL O_2_/kg/min) among adult (a) males and (b) females of the Igloolik Inuit, 1969–1970, 1979–1980, and 1989–1990, and of the Siberian Evenki and Ket, 1992. Across all age groups, the Indigenous Evenki and Ket have the lowest aerobic capacities. *Source:* Data from Rode and Shephard (1994b) and Katzmarzyk et al. (1994b).

The estimated aerobic capacities among the Evenki and Ket of Central Siberia in the early 1990s were notably lower than those of the Igloolik Inuit. Figure 13 compares the VO_2_max estimates of the Igloolik adults for all three study periods (from Rode and Shephard 1994b) to those of the Indigenous Evenki and Ket (from Katzmarzyk et al. 1994b). The Indigenous Siberian men and women have lower aerobic capacities than all of their Igloolik age peers. These results are striking and may partly reflect the impact of Soviet collectivization on the long‐term cardiorespiratory fitness of Indigenous Siberian populations.

Our work in Russia has also examined the impacts of the dramatic social and economic changes in post‐Soviet Russia on the health and well‐being of Indigenous Siberians. By the mid‐late 1990s, many of the Indigenous herding and farming cooperatives in Siberia were dismantled, resulting in a shift away from a subsistence lifestyle to a more market‐oriented lifestyle. Today, most families pursue a mixed economy, with some wage employment along with continued participation in some traditional subsistence‐based activities, such as herding, foraging, and/or farming in order to supplement their diets.

These once isolated, rural communities now have access to a wide variety of western products, producing shifts in food availability, energy expenditure, and cardiovascular disease risks. Of the sociodemographic measures we have looked at, the level of engagement in subsistence activities is consistently among the strongest predictors of biological and health outcomes.

**FIGURE 14:**
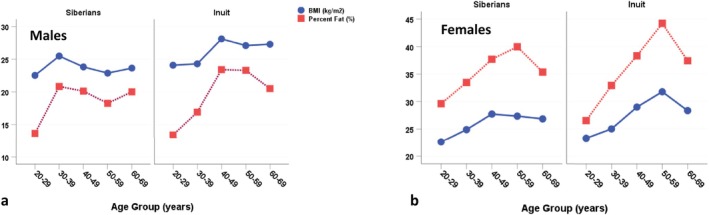
BMI (kg/m^2^) and percent body fatness (%) in Indigenous Siberian (Evenki, Ket, Buryat, Yakut) and Igloolik Inuit (a) males and (b) females ages 20–69 years. Across all ages, Siberian males have lower BMIs (23.7 vs. 26.2 kg/m^2^) and fatness (18.6% vs. 19.5%) than their Inuit counterparts. Among women, Inuit have greater BMIs (27.4 vs. 25.9 kg/m^2^), while the two groups are comparable in percent fatness (Siberians: 35.2%; Inuit: 35.8%). *Source:* Inuit data from Rode and Shephard (1994b), 1989–1990 survey. Percent body fatness estimated from sum of skinfolds using the Durnin and Womersley (1974) equations.

As observed among the Inuit, rates of overweight and obesity are also increasing among Indigenous Siberian populations (Snodgrass, Leonard, Sorensen, et al. 2006). Figure 14 compares BMI and percent body fat among indigenous Siberians (Evenki, Ket, Buryat, and Yakut) to those of the Igloolik Inuit measured in 1989–1990. Among men, Indigenous Siberians have lower BMIs (Siberians: 23.7 kg/m^2^; Inuit: 26.2 kg/m^2^) and percent fatness (Siberians: 18.6%; Inuit: 19.5%) than the Igloolik Inuit. Among women, the Inuit have higher BMIs (Siberians: 25.9 kg/m^2^; Inuit: 27.4 kg/m^2^), while the percent fatness is comparable between the two groups (Siberians: 35.2%; Inuit: 35.8%).

**FIGURE 15:**
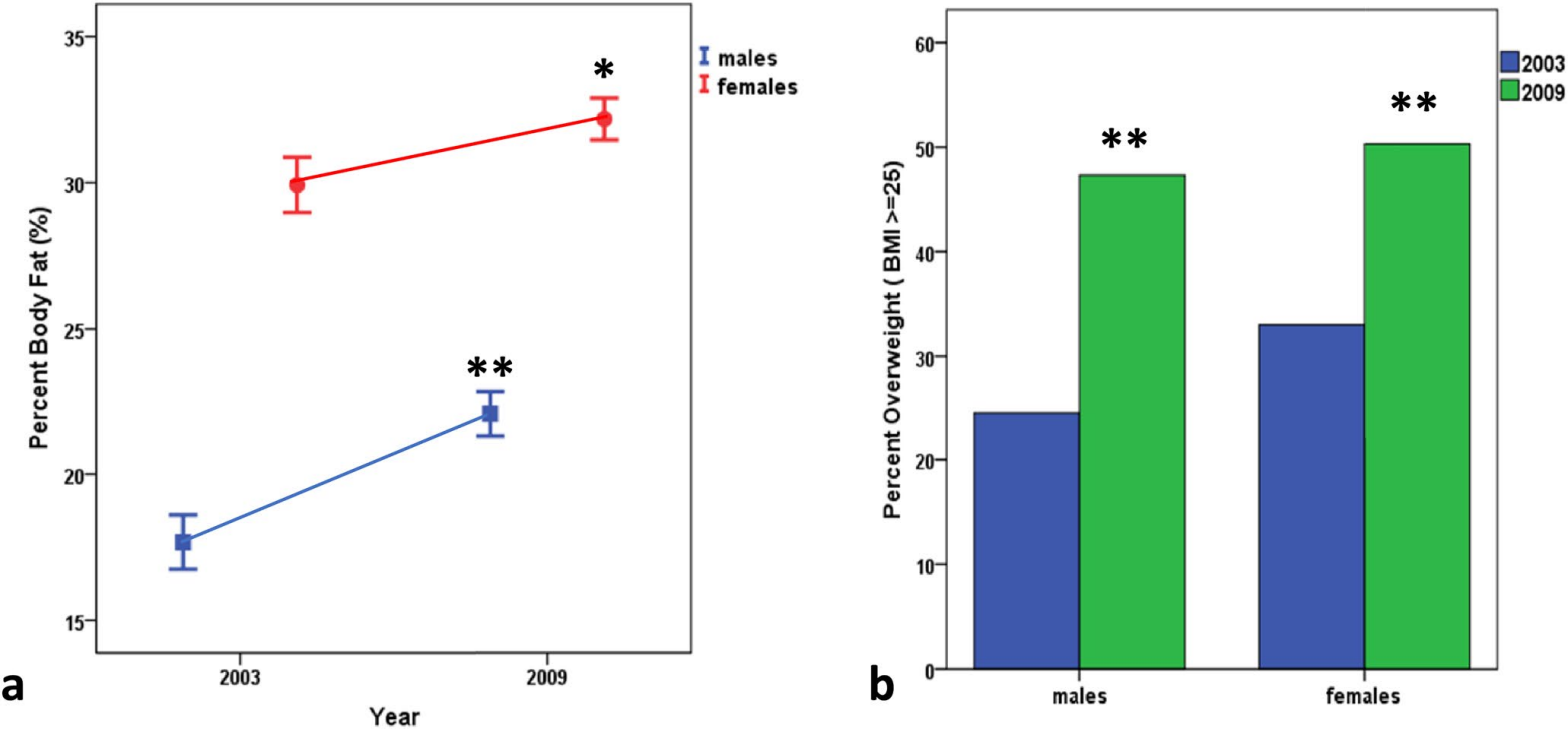
Trends in (a) percent (%) body fatness and (b) prevalence (%) of overweight and obesity (BMI ≥ 25 kg/m^2^) among Yakut adults between 2003 and 2009. Levels of adiposity and the prevalence of overweight and obesity have both significantly increased (*p < 0.05; **p < 0.01). *Source:* Adapted from Leonard (2024).

Figure 15 shows the consequences of lifestyle transitions among the Yakut. The graphs show changes in percent body fatness (Figure 15a) and the prevalence of overweight and obesity (Figure 15b) in the Yakut between 2003 and 2009. These changes closely parallel those documented by Shephard and Rode among the Igloolik Inuit.

However, in contrast to the conditions in Igloolik in the early 1990s, our work suggests that the preservation of traditional subsistence lifeways is still associated with greater activity and better health in the Yakut. Research by Josh Snodgrass examined the influence of subsistence participation on TEE and daily PALs as measured using the doubly labeled water technique in a sample of Yakut adults (Snodgrass, Leonard, Tarskaia, and Schoeller 2006). Greater participation in subsistence activities was associated with significantly higher levels of TEE and physical activity (*p* < 0.05). In contrast, those individuals who were most integrated into the market economy with little or no involvement in traditional activities had much more sedentary lifestyles and significantly lower levels of energy expenditure.

Thus, we find that the adoption of a more market‐oriented lifestyle is associated with reductions in TEE. This lifeway is also associated with greater risks of obesity and cardiometabolic health problems. Levy et al. (2016) have shown that among the Yakut, individuals with greater involvement in subsistence tasks had significantly less weight and fat gain; and significantly better measures of cholesterol, and systolic and diastolic blood pressure.

## 4 Conclusions

In sum, the extraordinary work of Roy Shephard and his colleagues among the Inuit of Igloolik provided the field of human biology with foundational insights into how human populations adapt to arctic climates, and how the transition to a market‐oriented lifestyle erodes fitness and metabolic health. Our work in Siberia has confirmed and expanded upon Shephard's findings in Igloolik, highlighting high levels of basal energy expenditure that are shared across circumpolar populations as well as the high levels of physical activity required for traditional subsistence life in the arctic. In Siberia, we are also seeing similar trends as in Igloolik of declining cardiometabolic health associated with the adoption of more sedentary lifeways. Together, these findings highlight the need for developing culturally specific strategies for promoting health across different populations.

Over the last 20 years, there has been growing interest in the study of circumpolar human biology and health, sparked in part by the dramatic social and environmental changes impacting this region (e.g., Galloway et al. 2011, 2010; Gildner and Levy 2021; Ocobock 2024; Ocobock et al. 2020, 2025; Sellers et al. 2023; Snodgrass et al. 2005, 2007; Young 2007). This innovative new research is providing a richer understanding of the biology of circumpolar populations and the distinctive health challenges that they face. All of this work builds upon the profound contributions of Professor Roy Shephard!

## Data Availability

Data sharing not applicable to this article as no datasets were generated or analysed during the current study.
